# A Temperature Imaging Method for Multi-Chip High Power LEDs Based on the Magnetic Nanoparticle Thermometer

**DOI:** 10.3390/nano12193280

**Published:** 2022-09-21

**Authors:** Zhongzhou Du, Bin Hu, Na Ye, Yi Sun, Haochen Zhang, Shi Bai

**Affiliations:** 1 School of Computer and Communication Engineering, Zhengzhou University of Light Industry, Zhengzhou 450001, China; 2 Department of Electrical and Electronic Engineering, Kyushu University, Fukuoka 6-10-1, Japan; 3 School of Information Science and Engineering, Shenyang University of Technology, Shenyang 110178, China

**Keywords:** magnetic nanoparticles, core size distribution, 2-D temperature imaging, multi-chip LED

## Abstract

In this study, a temperature imaging method based on a magnetic nanoparticle thermometer is proposed and evaluated. We first constructed a new model for finding the single temperature of magnetic nanoparticles with core size distribution. Specifically, we employed an air-core coil as a magnetic probe, which measured the magnetization of magnetic nanoparticles (MNPs). We then constructed a relation between the output signal of an air-core coil in the direction of the geometric center axis and the magnetization of the MNPs in a 2-D imaging area based on the magnetic dipole theory. Once this was achieved, we established a temperature imaging model by utilizing Green function as the convolution kernel, which describes the distance relationship between MNPs and the geometric center axis of the air-cored coil. After this, we calculated the harmonic distribution by a deconvolution algorithm and determined the temperature of the MNPs at different positions based on the model of harmonic amplitude and temperature, resulting in the 2-D temperature distribution. The simulation proved that the model and method of 2-D temperature distribution measurement could theoretically be acceptable. In the experiment, the 2-D temperature distribution of multi-chip power LEDs was measured accurately by a homemade system, thus demonstrating the feasibility of the proposed method for temperature imaging. This method is expected to provide a new solution for measuring the internal temperature distribution of opaque objects under extreme conditions.

## 1. Introduction

Temperature distribution is one of the most important parameters in biomedical and industrial fields, and is key in, for example, tumor hyperthermia [1,2,3], as well as in the construction of insulated-gate bipolar transistors (IGBT) [4,5,6] and high-power light-emitting diodes (LEDs) [7]. High-power integrated devices are widely applied to industrial applications. White light-emitting diodes (LEDs) [8,9,10] are used extensively in industrial applications, transportation, and in settings that are a part of normal daily life. The thermal management of LED is becoming increasingly problematic because increasingly powerful LEDs or LED arrays are being developed [11]. The heat accumulation causes the junction temperature to rise, thereby reducing the lifetime and luminous efficiency of the LED, and seriously affecting the stability of the LED array [12]. The ability to measure the real-time and precise temperature distribution is key to improving the multi-chip power LED’s lifetime and reliability.

Although many temperature measurement methods under routine conditions have been developed [13,14,15], it is difficult to accurately measure the internal temperature distribution of LEDs. Due to the limitations of the package structure, the junction temperature of the LED cannot be directly detected by physical-contact thermometry. Currently, several indirect or non-contact methods are available for junction temperature measurement, such as electrical methods based on forward voltage [16], reverse current [17], turn-on delay [18], etc., and optical methods based on peak wavelength [19], blue and white ratio (W/B) [20], center of mass wavelength of spectrum [21], relative reflected intensity [22], etc. However, the switching time of the current leads to a temperature error for electrical methods, and the temperature measured by optical methods is an average junction temperature of a whole LED. For the 2-D temperature distribution of LEDs, traditional infrared (IR) thermal imaging can provide up to 0.05 K temperature resolution [23]. However, the IR thermal imaging method can only measure the surface temperature distribution of the LED and not that of the junction. Although confocal micro-Raman microscopy is designed to measure the 3D temperature distribution of LEDs [24], the integrity of an LED cannot be guaranteed to be retained if micro-Raman microscopy is used to measure the LED temperature distribution, and micro-Raman microscopy cannot be used in all the environments in which LEDs may be used. Measuring the temperature distribution by micro-Raman microscopy requires the object being measured to be exposed to a laser for 10–20 s and gives a temperature resolution of 1–4 K. The currently available tools for measuring LED temperature distribution all have limitations; they cannot be used to accurately measure the internal LED temperature distribution in real-time or guarantee that the integrity of the LED is retained.

The magnetic nanoparticle thermometer [25,26,27], based on temperature sensitivity and nonlinear features of the magnetization response of magnetic nanoparticles (MNPs) induced by an applied magnetic field, is a noncontact and precise tool for measuring the internal temperature of objects. In the previous study, we utilized the magnetic nanoparticle thermometer to directly measure the temperature of the junction and the phosphor layer in white LEDs with one chip [28], which works under the assumption that the magnetic nanoparticles have an ideal core size without particle size distribution. Zhong et al. [29] proposed a method of magnetic nanoparticle temperature imaging using a scanning magnetic particle spectrometer. A phantom ‘E’ filled with an MNP sample of SHP-15 was used to perform the temperature imaging experiments. However, Brownian relaxation causes a phase lag between the magnetization of the MNPs and the ac magnetic field, resulting in a harmonic phase lag. Moreover, it is well known that the temperature distribution of MNPs with different core sizes widely differs in practical samples. The magnetization of MNPs is heavily dependent on core diameter and core size distribution [30] and cannot be described accurately by Langevin function when only one core size is taken into account. Assumptions based on one core diameter may introduce large temperature measurement errors. Consequently, we constructed a temperature model with core size distribution and used dry magnetic nanoparticles for the temperature imaging experiment. The dry magnetic nanoparticles sample will be mixed with silica gel while solidifying, and then the viscosity coefficient will become infinity, and the resulting Brownian relaxation time will be far beyond the experiment time scale.

In this paper, we reported a 2-D temperature imaging method that involves scanning using a magnetic nanoparticle thermometer, which is a non-destructive and precise technique for measuring the 2-D temperature distribution. The theory and experimental processes are described in detail. The temperature distribution model is established by utilizing Green function, constructed by measuring the distance between MNPs and the geometric center of the detection coil based on magnetic dipole theory. We employ mechanical devices to scan the 2-D imaging area coated with magnetic nanoparticles to obtain the first and third harmonic distribution, and then calculate the temperature of the MNPs at each point, resulting in the 2-D temperature distribution. The temperature of the substrate with no magnetic nanoparticles is obtained by heat transfer model. In the experiment, the internal temperature distribution of multi-chip power LEDs was successfully measured, thus demonstrating the feasibility of the proposed method for temperature imaging.

## 2. Model and Simulation

### 2.1. Temperature Model with Core Size Distribution

The magnetization of MNPs can be described by the Langevin function when the MNPs are exposed to a low-frequency excitation field. The ensemble magnetization *M* can be described as follows:

(1) $$M\left(t\right)=\phi M_{s}\int_{0}^{\infty }P\left(d_{c}\right)\left(\coth\left(\frac{mH_{ac}}{k_{B}T}\right)-\frac{k_{B}T}{mH_{ac}}\right)dd_{c}$$

where *P*(*d_c_*) = *n*(*d_c_*)*V*(*d_c_*) is the weight of core size distribution, *n*(*d_c_*) is the number of magnetic nanoparticles with core diameter *d_c_*, *V*(*d_c_*) is the volume of magnetic nanoparticles with core diameter *d_c_*, *ϕ* is the volume fraction of the MNPs sample, *H_ac_* = *H*_0_sin(*wt*) is the excitation, *H*_0_ is the amplitude of the excitation field, *w* = 2*πf* is the angular frequency, and *f* is the frequency, *k_B_* is Boltzmann’s constant, *T* is the absolute temperature, *M_s_* is the saturation magnetization, *m* = *M_s_V*(*d_c_*) is the magnetic moment.

The core size diameter *d_c_* of magnetic nanoparticles is given by discrete values to obtain a numerical solution to Equation (1) with the sampling points *K*, and then the ensemble magnetization *M*(*t*) can be rewritten as:

(2) $$M\left(t\right)=\phi M_{s}\sum_{k=1}^{K}P(d_{c,k})\left(\coth\left(\frac{m_{k}H_{ac}}{k_{B}T}\right)-\frac{k_{B}T}{m_{k}H_{ac}}\right)\Delta d_{c}$$

A Taylor series expansion of Equation (1) and the consolidation of similar items on a frequency basis allow for the 1st and 3rd harmonic amplitudes of ensemble magnetization *M* to be expressed as:

(3) $$\left\{\begin{array}{l} A_{1}=\phi M_{s}\left(\frac{\chi }{3T}-\frac{\chi^{3}}{60T^{3}}+\frac{\chi^{5}}{756T^{5}}-\frac{\chi^{7}}{8640T^{7}}\right)\\ A_{3}=\phi M_{s}\left(\frac{\chi^{3}}{180T^{3}}-\frac{\chi^{5}}{1512T^{5}}+\frac{\chi^{7}}{14400T^{7}}\right) \end{array}\right.$$

where $\chi =\sum_{k=1}^{K}P(d_{c,k})\frac{m_{k}H_{0}}{k_{B}}$.

The core size distribution can be obtained via VSM or measured by the ac harmonic [31]. Therefore, solving Equation (3) allows the temperature at a single point to be measured using the Levenberg–Marquardt algorithm.

### 2.2. Model for 2-D Temperature Distribution

An MNP is a superparamagnetic material that is sensitive to temperature and can be used to measure temperature [32]. The nonlinear magnetization $\vec{M}(\vec{R^{\prime }})$ of MNPs at the location $\vec{R^{\prime }}$ exposed to an applied magnetic field $\vec{H}$ can be described by the Langevin function, as shown in Equation (4),

(4) $$\vec{M}(\vec{R^{\prime }})=\phi M_{s}\left(\coth\left(\frac{m\vec{H}}{k_{B}T_{\vec{R^{\prime }}}}\right)-\frac{k_{B}T_{\vec{R^{\prime }}}}{m\vec{H}}\right)$$

where *ϕ* is the MNP volume fraction, *M_S_* is the saturation magnetization, $\vec{H}$ is the applied ac magnetic field, and $T_{\vec{R^{\prime }}}$ is the absolute temperature of magnetic nanoparticles at the location $\vec{R^{\prime }}$.

The air-core coil is employed for probing the magnetization of magnetic nanoparticles. The magnetic induction of the detection coil at a given spatial position $\vec{R}$ is given by

(5) $$\vec{B}(\vec{R})=\frac{\mu_{0}}{4\pi }\int_{s}\left(\frac{3\vec{M}(\vec{R^{\prime }})(\vec{R}-\vec{R^{\prime }})}{{\left|\vec{R}-\vec{R^{\prime }}\right|}^{5}}(\vec{R}-\vec{R^{\prime }})-\frac{\vec{M}(\vec{R^{\prime }})}{{\left|\vec{R}-\vec{R^{\prime }}\right|}^{3}}\right)ds$$

where *s* is the element plane.

**Figure 1:**
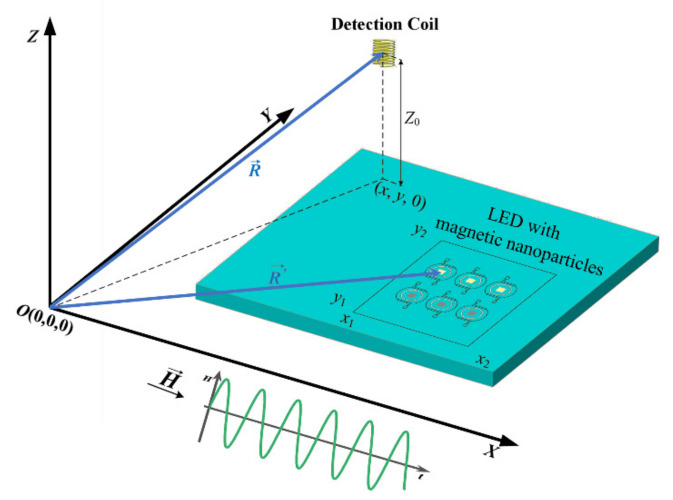
Configuration of the 2-D scanning setup. The 2-D MNPs distribution (a rectangle) is on the (*x*, *y*) plane. MNPs are exposed to a homogeneous ac magnetic field $\vec{H}$, perpendicular to the sensitive axis (*z*-axis) of the detection coil.

As shown in Figure 1, in Cartesian coordinates, magnetic nanoparticles are plotted on the xOy plane. The sensitive axis of the detection coil is parallel to the z-axis. The applied ac magnetic field, $\vec{H}$, is perpendicular to the sensitive axis of the detection coil, in order to prevent saturation and enable high dynamic range measurements. The magnetic moments of the MNPs are oriented along the x-axis, and the 2-D magnetization distribution induced by the ac magnetic field is denoted by $M\left(x,y,\phi ,T\right)$. For a given spatial position $\left(x,y,z_{0}\right)$ of the detection coil, a total magnetization *B_z_*, sensed by the detection coil along the z-axis, can be expressed in Equation (6) [32]:

(6) $$B_{z}=\frac{\mu_{0}}{4\pi }\int_{x_{1}}^{x_{2}}\int_{y_{1}}^{y_{2}}\frac{-3z_{0}(x-x^{\prime })}{{\left({\left(x-x^{\prime }\right)}^{2}+{\left(y-y^{\prime }\right)}^{2}+z_{0}^{2}\right)}^{5/2}}M(x^{\prime },y^{\prime },\phi ,T)dx^{\prime }dy^{\prime }$$

The magnetization induced by the applied ac magnetic field is composed of various harmonic components. For the *i*th harmonic of magnetization, the relationship between the total sum of harmonic amplitude $C_{i}(x,y,\phi ,T)$ at a given spatial position $\left(x,y,z_{0}\right)$, and the 2-D harmonic distribution, $A_{i}(x,y,\phi ,T)$, can be expressed by Equation (7):

(7) $$C_{i}(x,y,\phi ,T)=\frac{\mu_{0}}{4\pi }\int_{x_{1}}^{x_{2}}\int_{y_{1}}^{y_{2}}\frac{-3z_{0}(x-x^{\prime })}{{\left({\left(x-x^{\prime }\right)}^{2}+{\left(y-y^{\prime }\right)}^{2}+z_{0}^{2}\right)}^{5/2}}A_{i}(x^{\prime },y^{\prime },\phi ,T)dx^{\prime }dy^{\prime }$$

As shown in Equation (8), the measured magnetic induction is the convolution product of the magnetization $M\left(x,y,\phi ,T\right)$ of the MNPs with a weighting function usually called Green’s function $G\left(x,y\right)$ which depends on the distance of the MNPs to the magnetic sensor, such as

(8) $$G(x,y)=\frac{-3z_{0}(x-x^{\prime })}{{\left({\left(x-x^{\prime }\right)}^{2}+{\left(y-y^{\prime }\right)}^{2}+z_{0}^{2}\right)}^{5/2}}$$

In Equation (9), $C_{i}(x,y,\phi ,T)$ is measured by the detection coil. $A_{i}(x,y,\phi ,T)$ is an unknown to be determined. The relationship is shown as follows:

(9) $$C_{i}(x,y,\phi ,T)=G(x,y)\times A_{i}(x,y,\phi ,T)$$

A deconvolution algorithm enables one to measure the *i*th harmonics of the magnetization generated by the local MNPs at position P(x,y), expressed as

(10) $$A_{i}\left(x,y,\phi ,T\right)=F^{-1}\left(\frac{F\left[C_{i}\left(x,y,\phi ,T\right)\right]}{F\left[G\left(x,y\right)\right]}\right)$$

The condition number *N*(*G*) of matrix *G* acts as a measure of the ill-posed property. Assuming that *k*_1_ > *k*_2_ >. . .> *k_m_* denotes the m singular value of the SVD of matrix *G*, the condition number of *G* is the ratio of the maximum and minimum singular values:

(11) $$N(G)=\left\|G\right\|\cdot \left\|G^{-1}\right\|=k_{1}/k_{m}$$

However, attempts to seek the smallest singular value were fruitless due to quantization and limitations in machine accuracy. Accordingly, the most feasible method was to find the lower limit of the ‘genuine’ singular value, thus avoiding an oversized condition number.

The harmonic amplitudes at *ω* and 3*ω* are functions of the concentration ϕ and temperature *T*. Subsequently, the local temperature of MNPs at position P(x,y) can be calculated by Equation (8) under the applied ac magnetic field $\vec{H}=H_{0}\sin\left(\omega t\right)$. Solving Equation (8) allows the temperature to be measured using the Levenberg-Marquardt algorithm. After scanning the whole 2-D imaging area, the amplitudes of 1st and 3rd harmonics ($A_{1}(x,y,\phi ,T)$ and $A_{3}(x,y,\phi ,T)$) at each pixel point in the area of the image can be calculated by using a deconvolution algorithm. Consequently, combined with Equation (3), the local 2-D temperature distribution of the magnetic nanoparticles can be determined.

## 3. Simulation

**Figure 2:**
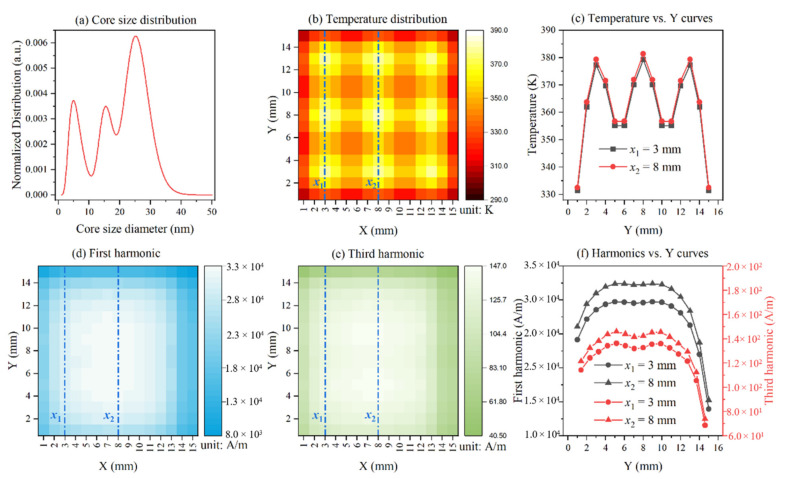
(**a**) The hypothetical core size distribution of MNPs. (**b**) The temperature distribution of 2-D imaging area coated with MNPs. The entire 2-D imaging area is 15 mm × 15 mm, with the resolution of 15 px × 15 px. (**c**) The temperature versus Y curves along the white dashed lines (*x*_1_ = 3 mm and *x*_2_ = 8 mm). (**d**) First harmonic distribution of magnetic nanoparticles on each pixel point after scanning. (**e**) Third harmonic distribution of magnetic nanoparticles on each pixel point after scanning. (**f**) The harmonics versus Y curves along the white dashed lines (*x*_1_ = 3 mm and *x*_2_ = 8 mm).

We performed some simulations to verify the feasibility of the 2-D temperature imaging method by scanning magnetic nanoparticles. The MNPs have a volume fraction of 1.5 × 10^−5^ and a saturation magnetization of 477 kA/m. For the distributions, we assumed that *P*(*d_c_*) obeyed a bimodal log-normal size distribution given by:

(12) $$P\left(d_{c}\right)=\sum_{k=1}^{3}\omega_{k}LN\left(d_{c};\mu_{k},\sigma_{k}^{2}\right)$$

(13) $$LN\left(d_{c};\mu_{k},\sigma_{k}^{2}\right)=\frac{1}{\sigma_{k}d_{c}\sqrt{2\pi }}\exp\left[-\frac{{\left(\ln d_{c}-\ln\mu_{k}\right)}^{2}}{2\sigma_{k}^{2}}\right]$$

where *μ* and *σ* are the mean and standard deviation, respectively. Table 1 lists the parameters for different MNP samples with bimodal log-normal distributions. Figure 2a indicates the hypothetical core size distribution of MNPs.

**Figure 3:**
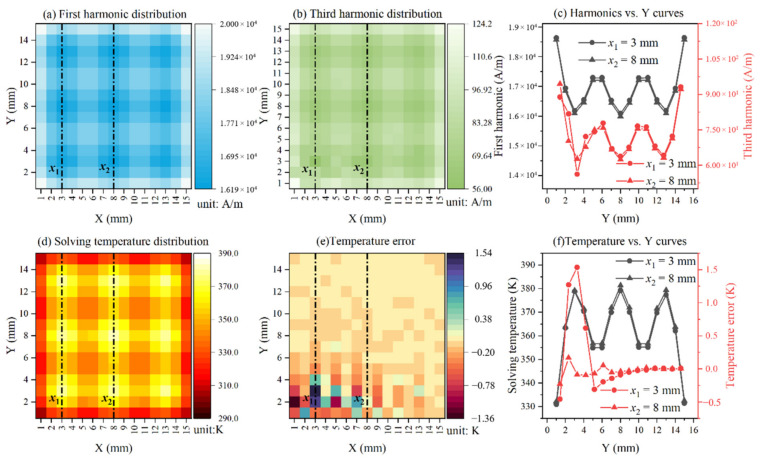
(**a**) First harmonic distribution of magnetic nanoparticles on each pixel point found using deconvolution algorithm. (**b**) Third harmonic distribution of magnetic nanoparticles on each pixel point was found using deconvolution algorithm. (**c**) The harmonics versus Y curves along the white dashed lines (*x*_1_ = 3 mm and *x*_2_ = 8 mm). (**d**) The solving temperature distribution of 2-D imaging area coated with MNPs. (**e**) The temperature error compared to the setting temperature. (**f**) The temperature versus Y curves along the white dashed lines (*x*_1_ = 3 mm and *x*_2_ = 8 mm).

First, the 2-D imaging area of 15 mm × 15 mm is assumed to be coated homogeneously with magnetic nanoparticles, with the size of each pixel point being 1 mm × 1 mm, making the resolution of the entire 2-D imaging area 15 px × 15 px. All of the 225 points (pixel point) are set at temperatures ranging from 290 K to 385 K as shown in Figure 2b,c, which show the temperature at positions of *x*_1_ = 3 mm and *x*_2_ = 8 mm. Then, an ac magnetic field with an amplitude of 4.6 kA/m and a frequency of 375 Hz is applied to the 2-D imaging area coated with MNPs. The detection coil, with 5 mm of vertical distance (*z*_0_) from the geometric center of the 2-D imaging area, is employed in order to measure the magnetization. We simulated the mechanical scan by changing the position of the detection coil. Figure 2d,e indicate the first harmonic and third harmonic amplitude distribution after scanning all pixel points in the 2-D imaging area. Figure 2f is the harmonic amplitude at positions of *x*_1_ = 3 mm and *x*_2_ = 8 mm. With these measurements taken, the amplitude of the first and third harmonic distribution can be obtained by Equation (7), as shown in Figure 3a,b. Figure 3c shows the harmonic amplitude at positions of *x*_1_ = 3 mm and *x*_2_ = 8 mm. Finally, the temperature of magnetic nanoparticles on each pixel point is calculated by Equation (8) using the Levenberg-Marquardt algorithm, resulting in the 2-D temperature distribution, as shown in Figure 3d. The temperature error compared to the setting temperature was shown in Figure 3e,f. The error increases with the increasing temperature; one possible explanation for this may be that the harmonic amplitude decreases with the increasing temperature. Overall, the simulated temperature error is acceptable.

## 4. Experiment

### 4.1. Experiment System

**Figure 4:**
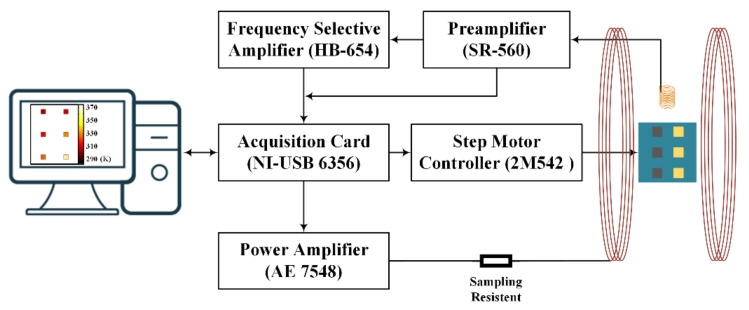
Sketch-view of the experimental setup used to measure the 2-D temperature distribution by scanning using a magnetic nanoparticles thermometer.

Figure 4 shows the 2-D temperature imaging system based on the magnetic nanoparticle thermometer, which is composed of an excited magnetic field generator, a magnetization measurement module, a mechanical scanner and a software module. The excited magnetic field is generated by the Helmholtz coil. First, the data acquisition card (DAQ) (NI-USB 6356, National Instruments, Austin, TX, USA) generates a sine signal, which is then inputted into the power amplifier (AE 7548, AE Techron, Elkhart, IN, USA) for amplification, causing the Helmholtz coil to generate the alternating excited magnetic field. The magnetization signal measurement module is composed of the air-core coil, preamplifier (SR-560, SRS, Sunnyvale, CA, USA) and frequency selective amplifier (HB-654, Hongbin Weak Signal Detection Co., Ltd., Nanjing, China). The magnetic nanoparticles in the 2-D imaging area generate the magnetization response under the excitation of the applied magnetic field. The magnetization of all magnetic nanoparticles is detected by the air-core coil, and is then amplified by the preamplifier. The magnetization response amplified by the preamplifier is secondarily amplified by the frequency-selective amplifier for the purpose of measuring the third harmonic. The mechanical scanner composed of the sliding table, step motor and step motor driver, is used to scan the 2-D imaging area coated with magnetic nanoparticles. Two step motors driven by 2M542 drivers (Leadshine, Shenzhen, China) will control the X-axis and Y-axis, respectively, of the sliding table, so that the samples can move in the 2-D area (the movement precision in one direction is 0.01 mm). After the whole temperature imaging area coated with MNPs is scanned, the amplified magnetization response measured will be collected by DAQ and fed into the software module for processing. The digital phase sensitive detection (DPSD) algorithm is used to calculate the amplitude of the first harmonic and third harmonic of the total magnetization response signals of the MNPs in the 2-D imaging area. The distribution of the first and third harmonics can be obtained using the deconvolution algorithm or by directly solving the system of linear equations. Then, the MNP temperature at different positions is determined by a model of harmonic amplitude and temperature, resulting in the 2-D temperature distribution.

### 4.2. Contrast Experiment with PT100 for Measuring Pixel’s Temperature

**Figure 5:**
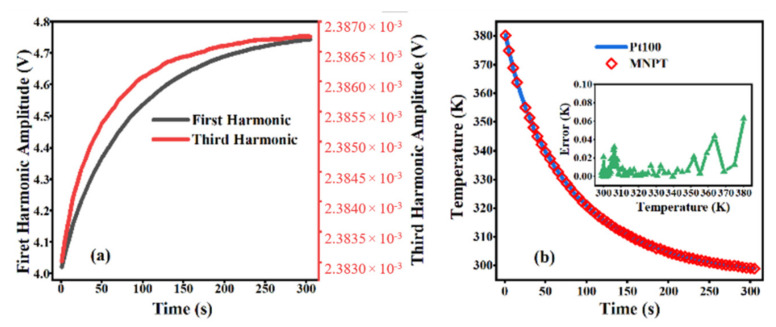
(**a**) The changing of harmonics in cooling of magnetic nanoparticles. (**b**) Temperatures were measured using the 2-D temperature imaging system and a Pt100 thermometer. The illustration is the temperature error between the 2-D temperature imaging system and a Pt100 thermometer.

To evaluate the performance and system stability of the 2-D temperature imaging system based on the magnetic nanoparticle thermometer, the single-pixel temperature measurement and comparison experiment also made use of a PT100 (calibrated using a 5627 A (Fluke Corporation, Everett, WA, USA, accuracy ± 0.025 K)). The MNPs used in this study were commercially available EMG1400 (FerroTec, Bedford, NH, USA, 10 nm, 360 kA/m). The magnetic nanoparticle particle samples were placed into the glass tube and heated to 390 K in the silicon soil. The glass tube containing the MNPs was then submerged in a mixture of ice and water to cool. The magnetic nanoparticle 2-D temperature imaging system and PT100 were used to measure the temperature change of the magnetic nanoparticle samples in cooling on a pixel-by-pixel basis. Figure 5a shows the changing trend of the first harmonic and third harmonic amplitudes during the cooling process of the magnetic nanoparticles. As shown in Figure 5b, the temperature trends measured using the magnetic nanoparticle 2-D temperature imaging system and the Pt100 thermometer were essentially the same. With the measurement results of the Pt100 as the benchmark, the measurement error of the magnetic nanoparticle 2-D temperature imaging system was less than 0.1 K.

### 4.3. 2-D Temperature Imaging Experiment

**Figure 6:**
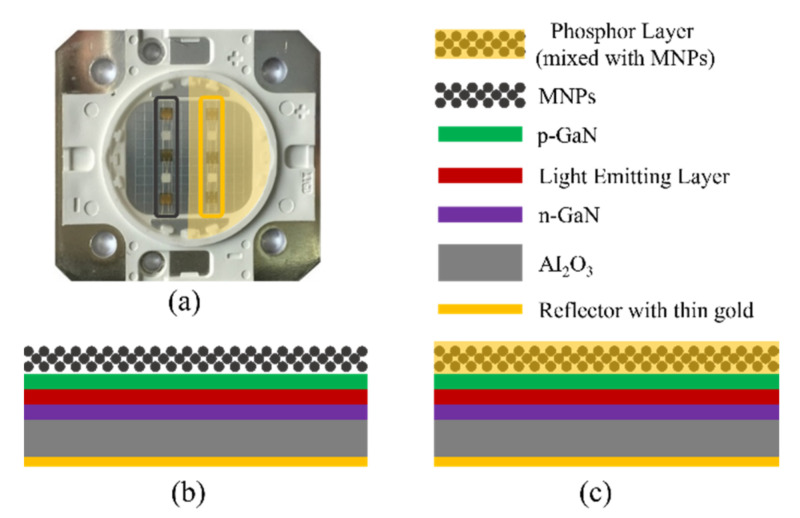
(**a**) Schematic of the multi-chip LED sample, (**b**) with magnetic nanoparticles coated directly on the top surface of LED chip (**c**) with MNPs mixed with the phosphor layer and coated on the LED chip.

In the previous study, we successfully measured the temperature of single-chip power LED chips and phosphor layers by using a magnetic nanoparticle thermometer [28]. We expect to measure the temperature distribution of multi-chip power LEDs by using the magnetic nanoparticle 2-D temperature imaging system in our study. The multi-chip power LED sample made use of two columns of LED chips (six LED chips in total). The magnetic nanoparticles were used to coat the LED chip and the phosphor layer. As shown in Figure 6, the magnetic nanoparticles were mixed with silica gel and then directly coated on the top surface of the LED chip for the three LED chips in the left column, so the measured temperature of the magnetic nanoparticles can be regarded as the temperature of the top surface of the LED junction/chip. The magnetic nanoparticle and the phosphor powder particles (YAG: Ce3 +) were embedded into the silica gel and then applied to the LED chip for the three LED chips in the right column, so the measured magnetic nanoparticle temperature can be thought of as the average temperature of the phosphor layer. The multi-chip LED sample was encapsulated by standard LED technology in transparent epoxy resin after curing at 150 °C for 3 h. We measured the optical power of the LED samples to evaluate the influence of the coat of MNPs on the optical performance using an integrating sphere spectroradiometer system (LHS-1000, Everfine, Shenzhen, China). Before and after the MNP coating, the optical powers were 857 mW and 850 mW, respectively, at 350 mA input, which indicates that the MNP layer fails to make a difference on the light extraction, and the temperature measured by the MNPs is the same as the practical LED temperature.

We first used the data acquisition card to generate a steady sinusoidal wave voltage signal with a frequency of 375 Hz and an amplitude of 5.0 V. The sinusoidal wave signal was amplified using a linear power amplifier, then directed through the low pass power filter and used to drive the Helmholtz coils to generate a steady ac magnetic field with an amplitude of 1.15 kA/m. The multi-chip LED sample containing the MNPs was placed in the test zone (the geometric center of the Helmholtz coil). For the power LED, the higher the excitation voltage is, the higher the heat generation power of the LED chip will be. Therefore, the higher the excitation voltage is, the higher the heat inside the LED will be under the same heat dissipation conditions, which means that the internal temperature is higher. To build a temperature gradient, we applied different excitation voltages on different chips inside the multi-chip LED samples. As shown in Figure 6a, six chips were deployed as three rows and two columns inside the multi-chip power LED. The magnetic nanoparticles were directly applied to the top surface of the chip (for the first column). For the second column, the magnetic nanoparticle and the phosphor powder particles were mixed and then applied to the top of the chip. The LED chips in the first, second and third rows were applied with an excitation voltage of 5.0 V, 5.1 V and 5.2 V, respectively. The magnetic nanoparticles inside the LED samples generated the magnetization response under the applied ac magnetic field. The magnetization response signals of the magnetic nanoparticles transform the magnetic signals to voltage signals via the air-core coil. After signals are amplified by the preamplifier, they are collected by the data acquisition card. The measurement time of the magnetization response of the single-point magnetic nanoparticle is 1 s. After the magnetization response of one point is measured, the mechanical device moves the air-core coil to the next point. It takes 11 s to scan and measure all points being tested.

**Figure 7:**
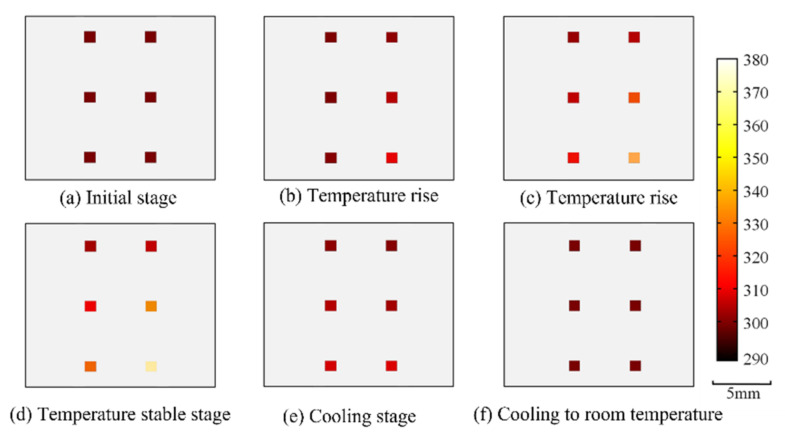
2-D Temperature distribution of multi-chip power LED in different stages (**a**–**f**).

Figure 7 shows the temperature distribution of the multi-chip power LED at different stages from the moment it is turned on, to the moment it is turned off. Since the temperature of the location with no magnetic nanoparticles cannot be measured, the temperature of the position is set at room temperature (298 K). Figure 7a shows the temperature distribution of the LED sample when the excitation voltage is not applied, with the entire measuring area at room temperature. Figure 7b,c show the temperature distribution of 400 s and 600 s, respectively, during the rising temperature stage. Figure 7d shows the internal temperature distribution of multi-chip power LED when the temperature stabilizes; the temperature of the first column chip coated directly with magnetic nanoparticles at an excitation voltage of 5.0 V, 5.1 V and 5.2 V is 305.1 K, 315.3 K and 328.2 K, respectively; the second column chip with the phosphor layer and magnetic nanoparticles at an excitation voltage of 5.0 V, 5.1 V and 5.2 V is 309 K, 333.5 K and 377.8 K, respectively. Under the excitation voltage of 5.0 V, 5.1 V and 5.2 V, the temperature of the fluorescence layer is 3.9 K, 18.2 K and 49.6 K higher than that of the chip. Figure 7e shows the internal temperature distribution of the multi-chip power LED as it cools, after the withdrawal of the excitation voltage. Figure 7f shows the temperature distribution of the multi-chip power LED when it is cooled to room temperature. In general, under different excitation voltages, the temperature of the fluorescence layer is higher than that of the chip; and the higher the excitation voltage, the larger the temperature difference between the two.

**Figure 8:**
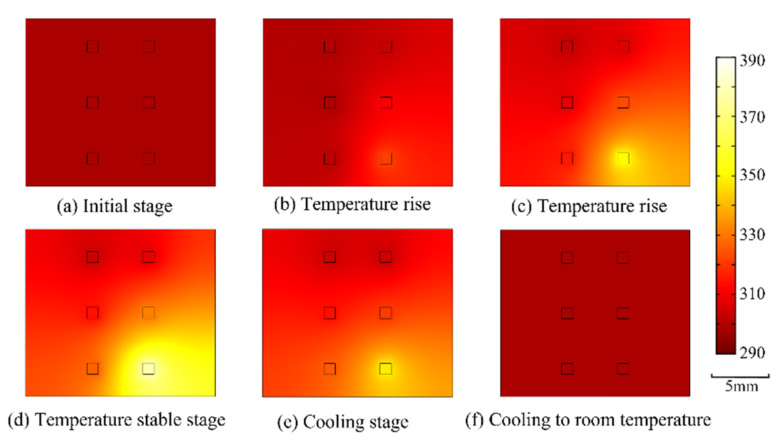
The temperature distribution of whole substrate in different stages (**a**–**f**).

The temperature of the substrate with no magnetic nanoparticle cannot be measured. As such, numerical methods are used to simulate the temperature distribution of substrate via COMSOL Multiphysics. The heat transfer model is given:

(14) $$\frac{\partial T}{\partial t}+\vec{v}\nabla T=\frac{k}{\rho C_{p}}\nabla^{2}T+\frac{Q}{\rho C_{p}}$$

where *ρ* is material density (kg/m^3^), *C_p_* is specific heat (J/(kg·K)), *k* is thermal conductivity (W/(m·K)), *T* is temperature (K), $\vec{v}$ is velocity vector (m/s) and *Q* is heat source (W/m^3^). The thermal conductivities of the LED chip, transparent epoxy resin and aluminum substrate are 200 W/m·K, 0.2 W/m·K and 10 W/m·K, respectively. The specific heat capacity of the LED chip is 0.49 J/g/K. Utilizing the chip temperature at different stages in Figure 7, the temperature distribution of the whole substrate can be obtained. As shown in Figure 8a–f, the region with the highest temperature is always the area surrounding the chip in the lower right corner at the stage when the LED sample is illuminated, because this chip was covered with phosphor powder particles and worked at a higher excitation voltage (5.2 V), causing it to generate a lot of heat.

## 5. Results and Discussion

The white LED absorbs light from the blue LED through a phosphor powder and generates heat through non-irradiated electron transitions. The phosphor powder particles are encapsulated in the silica gel layer whose lower thermal conductivity prevents the heat of the phosphor powder from dissipating, which means more heat will be accumulated in the phosphor layer and the fluorescence layer will be hotter than the chip. The cycle of scanning the whole measurement area is 11 s in the study, and we assume that the temperature of each chip changes very little during the 11 s. The measurement cycle should be shortened for any measured objects whose temperature changes quickly. The measurement cycle is mainly constrained by the following factors. (1) The area to be measured. The area to be measured of the whole multi-chip power LED is 12 mm × 15 mm; if the area is large, the scan time for completing a measurement area will also be longer. (2) The density of the MNP sample. In this study, the multichip power LED integrates six chips in three rows and two columns; it is necessary to increase the laying density of the magnetic nanoparticles in order to get a more comprehensive temperature distribution. (3) The measurement time of the single-point magnetization signal. The harmonic amplitude is used to reflect the temperature in this study. The more abundant the magnetization response signal, the closer the value obtained using the DPSD algorithm will be to the actual value of the harmonics, and the more accurate the temperature obtained by the inversion will be; it is necessary to choose the appropriate measurement time of the magnetization response signal to improve the accuracy of temperature measurement. However, increasing the measurement time of the single-point magnetization response signal to improve temperature measurement accuracy will inevitably affect the scan time of the whole measuring area. (4) The moving speed of the mechanical device. In this study, the moving speed of the single-direction axis of the mechanical device is 5 mm/s. it is possible to reduce the scan time of the whole measuring area by increasing the moving speed, which will inevitably challenge the accuracy of the movement.

## 6. Conclusions

In this study, we reported a 2-D temperature imaging method involving scanning with a magnetic nanoparticle thermometer. The method developed is a non-destructive and precise means of measuring the 2-D temperature distribution based on magnetic nanoparticles. The theory and experimental processes were described in detail. We employed mechanical devices to scan the 2-D imaging area coated with magnetic nanoparticles to obtain the temperature distribution and successfully measured the internal temperature distribution of multi-chip power LEDs, thus demonstrating the feasibility of the proposed method for temperature imaging. The 2-D temperature imaging method which involves scanning with a magnetic nanoparticle thermometer is expected to be accurate, and fundamental for the thermal management and package optimization of multi-chip high-power LEDs, making them more applicable to different applications.

## Figures and Tables

**Table 1:** Parameters of hypothetical core size distribution for MNPs.

Symbol	*k* = 2	*k* = 2	*k* = 3
Weight *ω_k_*	0.2	0.2	0.6
Geometric mean *μ_k_* (nm)	6	15.8	25.5
Geometric standard deviation *σ_k_*	0.4	0.15	0.15
